# Modulating the Work Function of Graphene by Pulsed Plasma Aided Controlled Chlorination

**DOI:** 10.1038/s41598-018-35668-x

**Published:** 2018-11-26

**Authors:** Hiroshi Takehira, Mohammad Razaul Karim, Yuta Shudo, Masahiro Fukuda, Tsutomu Mashimo, Shinya Hayami

**Affiliations:** 1 Department of Chemistry, Graduate School of Science and Technology, Kumamoto University, 2-39-1 Kurokami, Kumamoto, 860-8555 Japan; 2 Department of Chemistry, School of Physical Sciences, Shahjalal University of Science & Technology, Sylhet, 3114 Bangladesh; 3 Institute of Pulsed Power Science (IPPS), Kumamoto University, 2-39-1 Kurokami, Chuo-ku, Kumamoto, 860-8555 Japan

## Abstract

Chlorine on graphene (G) matrices was doped by pulsed plasma stimulation on graphite electrode submerged in organochlorine solvents (CH_2_Cl_2_, CHCl_3_, CCl_4_). The study of work function by Kelvin probe force microscopy (KPFM) measurement clearly indicates that Cl-doped G behave like semiconductor and GG@CHCl_3_ exhibits the lowest value for the work function. We propose that this report not only represents a new route for tuning the semiconductivity of G but also indicates that doping level of halogen on G based carbon framework can be controlled by pulsed plasma treatment of carbon materials on various organohalogen derivatives.

## Introduction

Semi-conductive nature of graphene (G) quantified by work function has been studied herein. We expected that the thermodynamic work function of G surface can be modulated by doping a second element or generation of defects or hole at the graphitic carbon skeleton. Though the zero band gap of G indicates its ballistic transport behavior, some electronic application is necessarily associated with the opening of band gap with controlled electron conduction property and gated semiconducting behavior. In addition, the realm of organic electronics demands the development of flexible, transparent and conductive electrodes, which partly can be full filled by modification of G^1^. Even in the present decade, ITO ensures its application with high light transparency (>80%) and low resistance (10 to 30 Ω/cm), its inhomogeneous surface and instability in rough condition remains the major drawback for micro-electronic application^2^. Out of ITO, carbon nanotubes, metal nanowires and some organic conducting polymers including poly (3, 4-ethylenedioxythiophene)-poly(styrenesulfonate) have also been used in flexible electronics^3^. Though G is expected to be the best candidate in this realm, modulating the conductivity of G to some desired extent remains still tough. The difficulty of controlled chemical functionalization or doping on G surface is the major reason for this inconvenience. Though, ensuring some harsh condition, G can be chemically functionalization to engineer the band gap, it affects some other necessary properties including light transparency, stability and sheet-like nature^4^. To avoid this unwanted situation, G based electrodes generally are fabricated from some indirect routes, where graphene oxide (GO) obtained from graphite by various oxidation techniques are reduced to form reduced Graphene Oxide (rGO) by means of chemical or thermal treatment^5^. The rGO exhibits properties similar to G. The band in rGO is open due to the existence of some residual oxygenous sites and doped elements^6^. Conventionally, pristine G obtained from chemical vapor deposition is used in light-emitting diodes^7^, photovoltaics^8^, field-effect transistors^9^, and photo-detectors^10^. As elemental doping affects the band gap and conductivity of rGO or G, instead of a single synthetic process, various routes to obtain G or rGO is preferred to tune and adopt the semiconductivity to the desired applications. Besides opening the band gap, chemical modification to G results in the defect and vacancy creation, for which this G can be used for magnetism, fluorescent and gas storing systems. Hence, finding routes for doping G by a second element has attracted worldwide concern.

In this work, we have succeeded chlorination of G by liquid pulsed plasma method, where graphite electrode was submerged and exfoliated in various organochlorine solvents. The exfoliation process along with doping was aided by a periodic high voltage stimulation^11^ for very short time. The method has been preferred for some advantages including (i) the ratio of chlorination was possible to be controlled only by changing the solvent having varying percentages of chlorine, (ii) compared with some established method, the doping and exfoliation time has found to be much lower (iii) large-scale synthesis of doped G was possible. A series of doped G, with a varied amount of chlorine dopant and band gap, was obtained by using CH_2_Cl_2_, CHCl_3_ and CCl_4_ as the solvent.

The synthesis of halogen-doped G has been investigated in recent years. G modified by chloride or fluoride possess high electronegativity and was found to exist with vacancy and chemical reactivity. Generally, chlorination or fluorination of G is accomplished by exposing with Cl_2_ or F_2_ plasma, photochemical reaction and microwave assisted reaction. However, wide application of these methods is limited due to the necessity for exclusive experimental condition and failure in large-scale synthesis. Moreover, there still doesn’t exist any route to control the level of halogen doping on G. Bearing these issues, herein we have studied improved pulsed plasma-aided chlorination of G in organochlorine solvents. The generated G was modified by control over the doping level and tunability of thermodynamic work function. We propose that these improvements are highly desirable for practical applications of G in sensor and other devices.

## Results and Discussion

**Figure 1:**
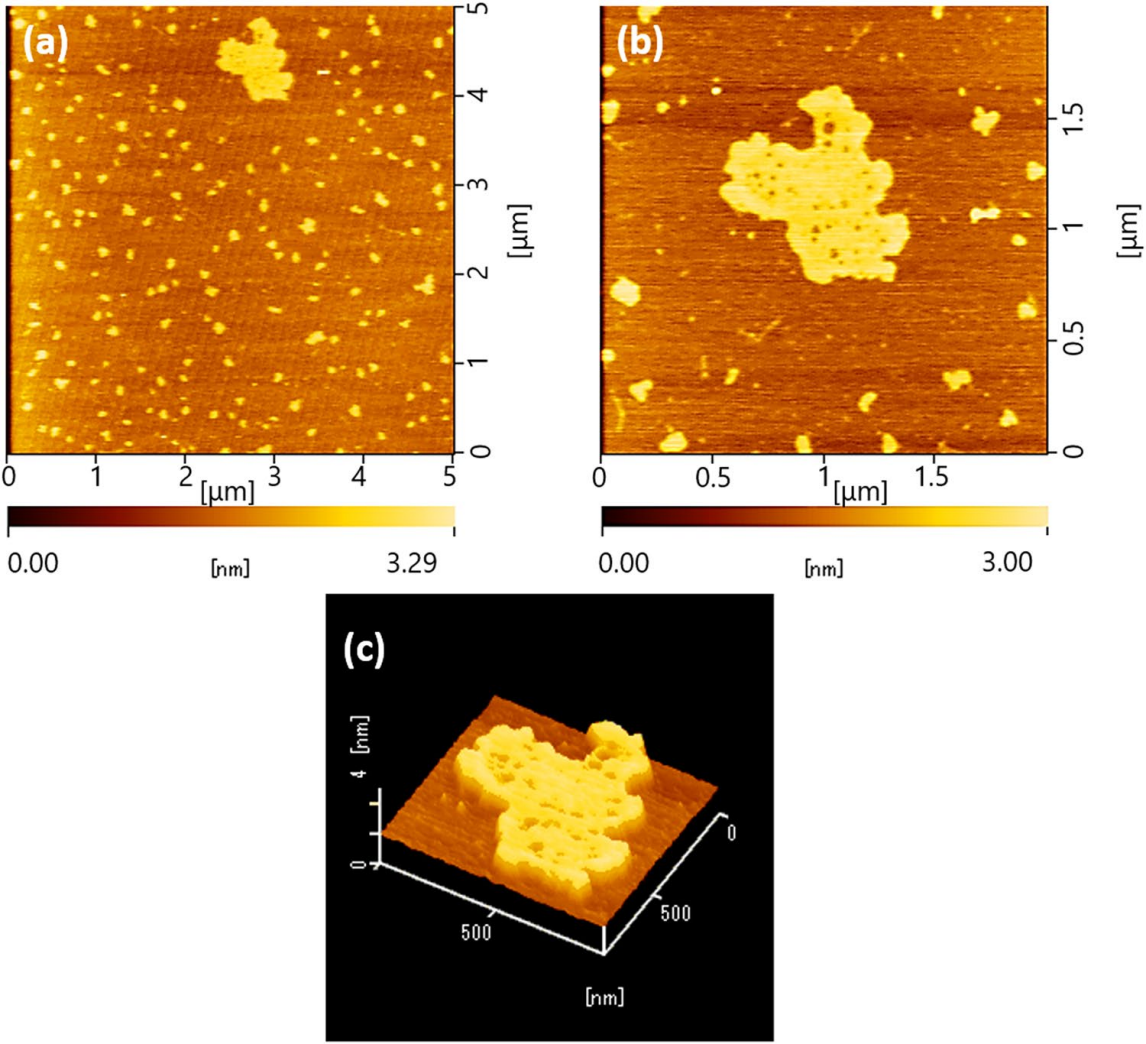
AFM image for GG@H_2_O shows the presence of G nanosheet with varied dimensions (**a**,**b**). The thickness is below 3 nm (**c**).

Figure 1 represents the AFM image of G nanosheets collected from aqueous suspension. Displayed morphology confirms the exfoliation of graphite rod into tiny nanosheet, which is G. The exfoliation is aided by pulsed plasma. Such exfoliation of graphite is commonly known to be assisted by any type of ionic intercalation^12^. The nanosheets seem to have a submicrometer dimension with the thickness below 3 nm.

**Figure 2:**
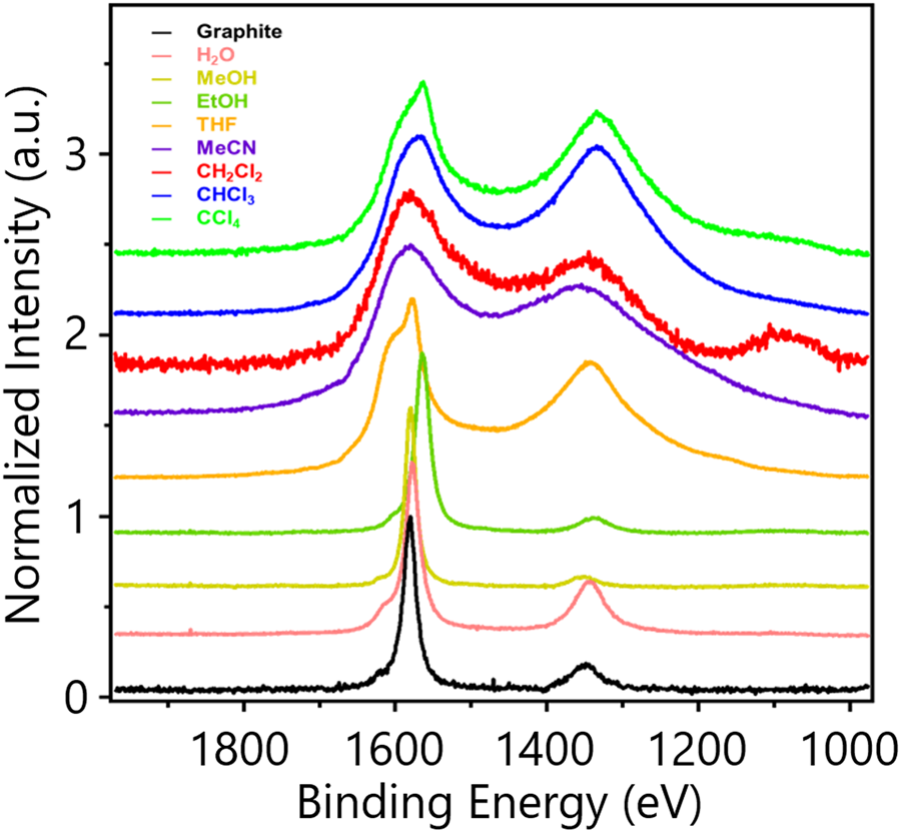
Raman spectra of Graphite and various G synthesized by submerging graphite rod in various solvents. Pulsed Plasma method.

Figure 2 represents the Raman spectra of pristine graphite rod, GG@CH_2_Cl_2_, GG@CHCl_3_ and GG@CCl_4_ and some other G samples collected from various colloidal suspensions generated from similar pulsed plasma experimentation in different solvents. For pristine graphite sample, two typical peaks of carbon materials namely the ‘D band’ (~1350 cm^−1^) and the ‘G band’ (~1580 cm^−1^) are observed. These two peaks are reported to be associated with the breathing mode (A_1g_) and the in-plane bond stretching motion of C sp^2^ atoms (E_2g_), respectively^13^. The D band implies the sp^3^ carbon sites, with edge and defects. Raman spectra of G is very specific, where a 2D band (~2650 cm^−1^) is derived from a number of layer of G. The G band position shifts slightly from 1579 cm^−1^ to 1580 cm^−1^ for the GG@CH_2_Cl_2_ sample. This inverse softening is a result of the change in electronic structure during exfoliation as the van der Waals stacking force is relaxed due to the exfoliation process. However, this hardening process is reverse to the softening explained in some previous reports^14^. The ratio of peak height (I_D_/I_G_) for D and G-band was calculated as 0.337 and 0.641 for graphite rod and GG@CH_2_Cl_2_, respectively. Though this difference corresponds to a 33% increase, the I_D_/I_G_ value clearly indicates the existence of G after the rod being exfoliated. Although electrochemical exfoliation with aqueous solvent is often utilized, some oxidation was observed in previous reports^15^. The I_D_/I_G_ value increasing for GG@CHCl_3_ and GG@CCl_4_ as 0.942 and 0.993 indicates the increase in chlorine content of the samples. We propose that chlorination at some carbon sites and generation of defects are responsible for such increase in I_D_/I_G_ value. Reported that the I_D_/I_G_ value is inversely proportional to the percentage of sp^2^ domain^16^. For GG@CH_2_Cl_2_, GG@CCl_3_ and GG@CHCl_3_, we also measured FT-IR spectra (Figure S1). Each sample shows gradual peak for C-Cl bond between 700 and 800 nm.

Therefore, the increase in this value is the result of decrease in sp^2^ domain and generation of some sp^3^ sites^17^. For observing the effect of solvent on exfoliation, we also accomplished the experiment using a series of solvents including EtOH, MeOH, H_2_O, THF, ACN and toluene. In every case, we observed the generation of colloidal dispersion with observable changes in Raman spectra. In a previous study, we have found that the increase in sp^3^ carbon site sometimes is associated with the generation of epoxy groups at G basement^18,19^. Though there exists no scope for such oxidation, to justify this possibility we have performed the chemical structure analysis by XPS study.

**Figure 3:**
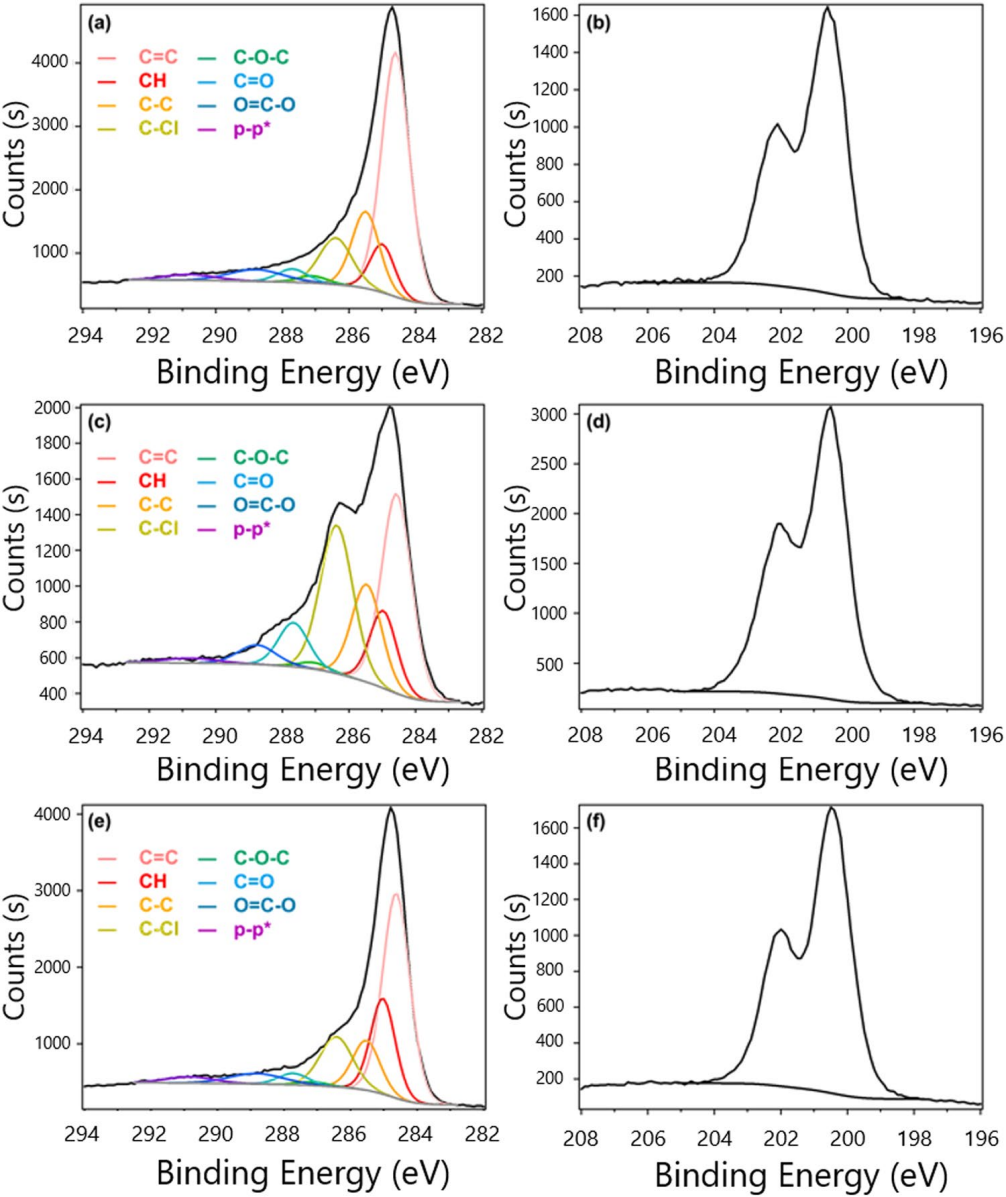
C_1s_ (**a**,**c**,**e**) and Cl_2p_ (**b**,**d**,**f**) spectra in XPS: GG@CH_2_Cl_2_ (**a**,**b**), GG@CHCl_3_ (**c**,**d**) and GG@CCl_4_ (**e**,**f**).

Figure 3(a,c,e) represents the C1s spectra of GG@CH_2_Cl_2_, GG@CHCl_3_ and GG@CCl_4_ in XPS. The spectra clearly indicate that the chemical structure of the nanosheet is graphene analogous. Possibilities for the formation of oxidized G was justified by de deconvolution of the envelop peak. The deconvolution spectrum for C=C (284.5 eV), C-C (285.0 eV), C-H (285.4 eV), C-OH (286.4 eV), C-O-C (287.2 eV), C=O (287.7 eV) and O=C-OH (288.7 eV) are shown in the figure, from which it is clear that the oxygenated functional groups have insignificant contribution to the envelop spectra. The spectra for C-Cl bonding in C1s spectra was overlapped with the spectra for C-O-C group. Hence, the deconvolution peak could be carried out with three features centered at 284.6, 285.8 and 287.6 eV corresponding to sp^2^ hybridized carbon, sp^3^ hybridized carbon and C–Cl respectively^20^. Figure 3(b,d,f) show the XPS Cl2p spectra of GG@CH_2_Cl_2_, GG@CHCl_3_ and GG@CCl_4_. The peak of Cl 2p_3/2_ and Cl 2p_1/2_ attributed to C-Cl bonding is observable around 200.4 and 202.2 eV. The peak attributed to Cl- anion (the peak of Cl 2p_3/2_ and Cl 2p_1/2_ is about 197.8 and 199.4 eV) is hardly present^21,22^. In addition, according to the semi-quantitative analysis of XPS, the ratio of C-Cl bonding in Cl2p spectra is almost equal the ratio of the peak at 286.4 eV (C-OH, C-Cl) in C1s spectra These results indicate that the peak at 286.4 eV in C1s spectra raises for the presence C-Cl bonding. The existence of C-Cl bond also is confirmed from elemental ratio obtained from XPS analysis. The ratio of Cl/(C + Cl) in GG@CH_2_Cl_2_, GG@CHCl_3_ and GG@CCl_4_ are 9.8, 29.7 and 12.6, respectively. The ratio of Cl/(C + Cl) for GG@CHCl_3_ is much than that for other samples. From the XPS survey spectra, GG@CHCl_3_ contained Cl 24 at.% (Figure S3b). This Cl contents is much higher compare to previous data^23,24^. Moreover, this Cl contents is one of the highest contents compare with other halides^24–26^.

**Figure 4:**
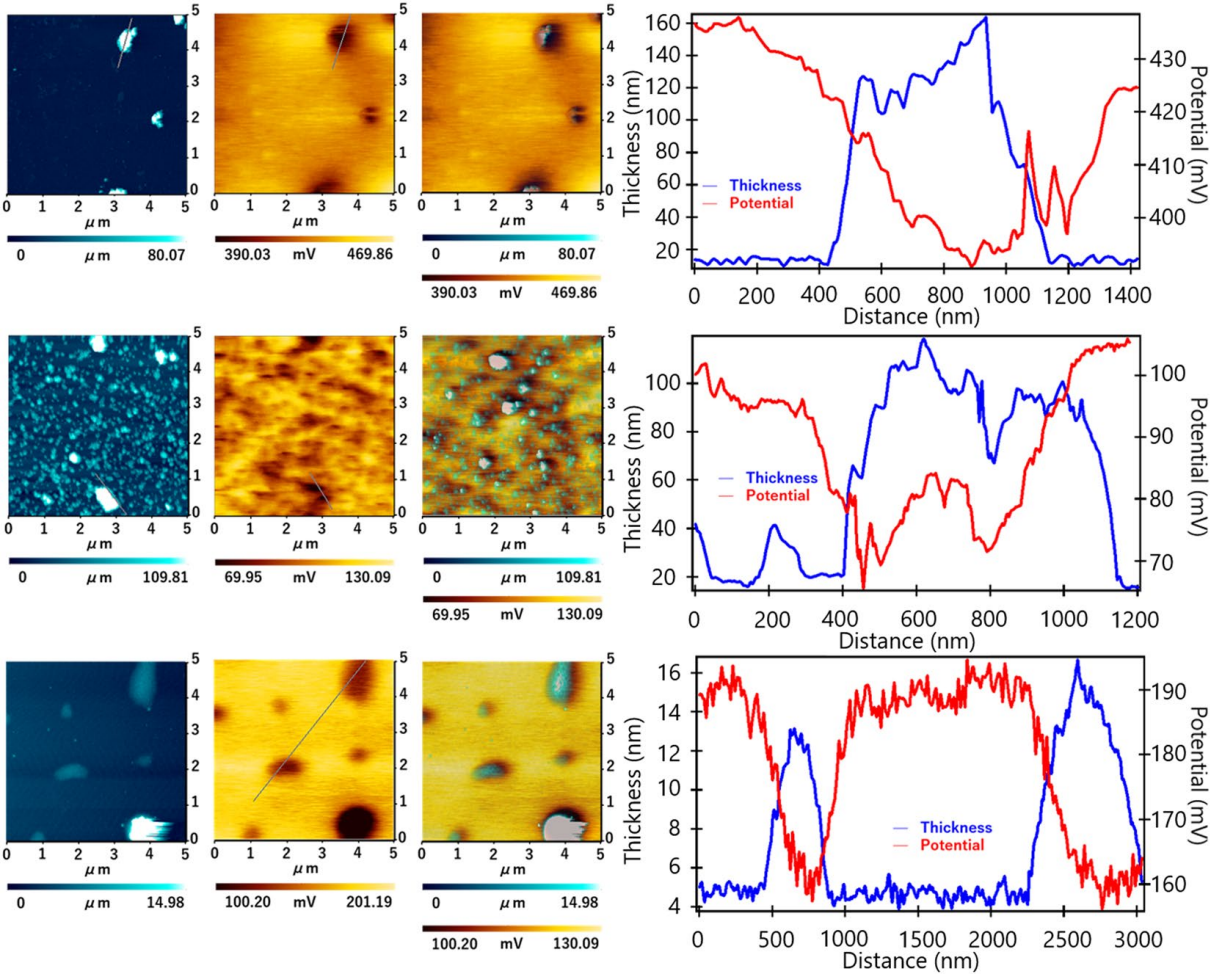
Study of Kelvin Probe Force Microscopy (KPFM) image and contact potential difference (CPD) profiles of the samples. Height profiles as (**a**,**e**,**i**); potential energy mappings as (**b**,**f**,**j**); superimposed topography for heights and CPD as (**c**,**g**,**k**) and superimposed curves for heights profiles and CPD as (**d**,**h**,**l**) are presented for GG@CH_2_Cl_2_, GG@CHCl_3_ and GG@CCl_4_, respectively.

Work functions of the sample surfaces were studied by Kelvin Probe Force Microscopy (KPFM)^27–29^. We have investigated the change in work function of G due to the variation in the extent of chlorination. KPFM measurement resulted in the CPD with respect to the height profile of the films. CPD quantifies the difference of work functions between the sample surface and the probe tip when they are in thermodynamic equilibrium. Hence, CPD analysis is very useful to confirm semiconducting properties of nanomaterials. A lower CPD value signifies higher work functions. We accomplished simultaneous imaging of topography and contact potential difference. Figure 4 shows the surface profiles, potential diagrams, superimposing images and CPD profile for each of GG@CH_2_Cl_2_, GG@CHCl_3_ and GG@CCl_4_. The surface profiles of the samples show that their morphologies are same as was estimated by TEM image analysis. The image for two-dimensional height profiles in Fig. 4(a,e,i) shows the nanometer ranged thickness of GG@CH_2_Cl_2_, GG@CHCl_3_ and GG@CCl_4_. Respective, potential energy mappings presented in Fig. 4(b,f,j) clearly indicate the topographical dependence of CPD throughout the surfaces. The superimposed mapping for height profile and CPD are presented in Fig. 4(c,g,k). Figure 4(d,h,l) display the superimposed curves for variation in both the sample heights and CPD as a function of scanned locations. CPD recorded for GG@CH_2_Cl_2_, GG@CHCl_3_ and GG@CCl_4_, lie within the range of 390.03-469.86, 69.95-130.09 and 100.20-201.19 mV, respectively. The CPD recorded for GG@CHCl_3_ is lower than CPD of other samples and hence the work function of GG@CCl_3_ is assigned to be the maximum. These results might be originated from the variation in the extent of chloride-modified domains in G basement.

G is composed of sp^2^ hybrid carbon atoms with perpendicularly lying p_z_ orbitals engaged in π bond network system. The large π system can participate in molecular charge-transfer process with both the electron donor and acceptor molecules/domains^30,31^. Such charge-transfer phenomena assist the formation of C-Cl bond during the pulsed plasma treatment of graphite in solvent. We propose that the bond formation is accomplished by simultaneous exfoliation of graphite with the formation of carbon and chlorine free radicals, which initiate the C-Cl bond formation. Therefore, remarkable changes in the Raman spectrum of G is noticed. Though the scope of oxidation during the reaction is very limited, the I_D_/I_G_ ratio increases due to the effect of doping and vacancy generation. The pulsed plasma-aided vacancy generation also could function for modulating the work functions of the samples. The addition of chloride is analogues to the previously reported hydrogenation of G, where sp^3^ C–H bond formation in a reversible way was utilized for the chemical storage system of hydrogen gas^32^. In a similar study, reversible fluorination of G was also found to be convenient for gas storage^33–35^. Chlorination of G by 56 wt.% (30 at.%) in liquid chlorine medium was passible to carry out by UV light irradiation^28^. The chlorinated sample decomposes on heating or on laser irradiation, releasing all the chlorine. Almost similar results were observed for the bromination of G.

Any type of structural interruption can affect the band structure of G. Therefore, the theoretical zero band gap of G becomes opened due to elemental doping, distortion on edges or simply the creation of vacancies. The intrinsic electron mobility in G is greatly affected by its interaction with the environment, dopant and even with the substrate^29^. In every case the formation of ‘disorder potential’ results in the reduction of mobilities and limiting energy gaps in nanoribbons and bilayer G^26^. The doping of chlorine on G has revealed such usual change in work functions. In addition, the possibility of the existence of some substrate-induced electron-hole puddles, doping domains and local quatum capacitances may have some miner effects^31^. KPFM experimentation with single and few-layer graphene was reported previously^32^. The work function of single and bilayer G can be modulated with respect to the carrier density and doping-induced carrier concentration. Shifting of Fermi level reveals such effects.

The work function is a fixed characteristic of surface and signifies optimized thermodynamic energy/work necessary for releasing an electron from a spot anywhere of the solid surface to a vicinity outside the surface by atomic scale distance. This surface property is associated with the estimation of semiconducting behavior as in semiconductors, the conduction bands are adjacent enough to the Fermi level with having the option for thermal energy driven population of electrons or holes. Therefore, the samples seem to have bandgap of semiconductor due to the doping of chlorine at the carbon skeleton of G.

## Conclusions

A series of chlorine doped G namely GG@CH_2_Cl_2_, GG@CHCl_3_ and GG@CCl_4_ has been synthesized by pulsed plasma treatment of graphite electrodes submerged in the organochlorine solvents including CH_2_Cl_2_, CHCl_3_ and CCl_4_, respectively. The samples were characterized by AFM image, which confirmed the sheet-like structure. The Raman spectra confirmed the increase in I_D_/I_G_ value of the chlorine doped G samples. This increment confirmed the destruction of sp^2^ carbon site and formation of sp^3^ carbon atoms due to the formation of the C-Cl bond. Finally, the XPS spectra confirmed the formation of G, with some C-Cl bonds, where the Cl/(C + Cl) ratio in GG@CH_2_Cl_2_, GG@CHCl_3_ and GG@CCl_4_ were 9.8, 29.7 and 12.6, respectively. The Thermodynamic work functions of the samples were measured by Kelvin Probe Force Microscopy (KPFM). Measurement of contact potential difference (CPD) by KPFM shows that CPD values for GG@CH_2_Cl_2_, GG@CHCl_3_ and GG@CCl_4_, lie within the range of 390.03–469.86, 69.95–130.09 and 100.20–201.19 mV, respectively. The work function clearly indicates that all the samples behave like semiconductor. GG@CHCl_3_ exhibits the lowest value of work function. We propose that this report represents a new route for tuning the semiconductivity of G. Besides, the doping level of halogen on G is expected to be modulated by pulsed plasma treatment reported herein.

## Methods

**Figure 5:**
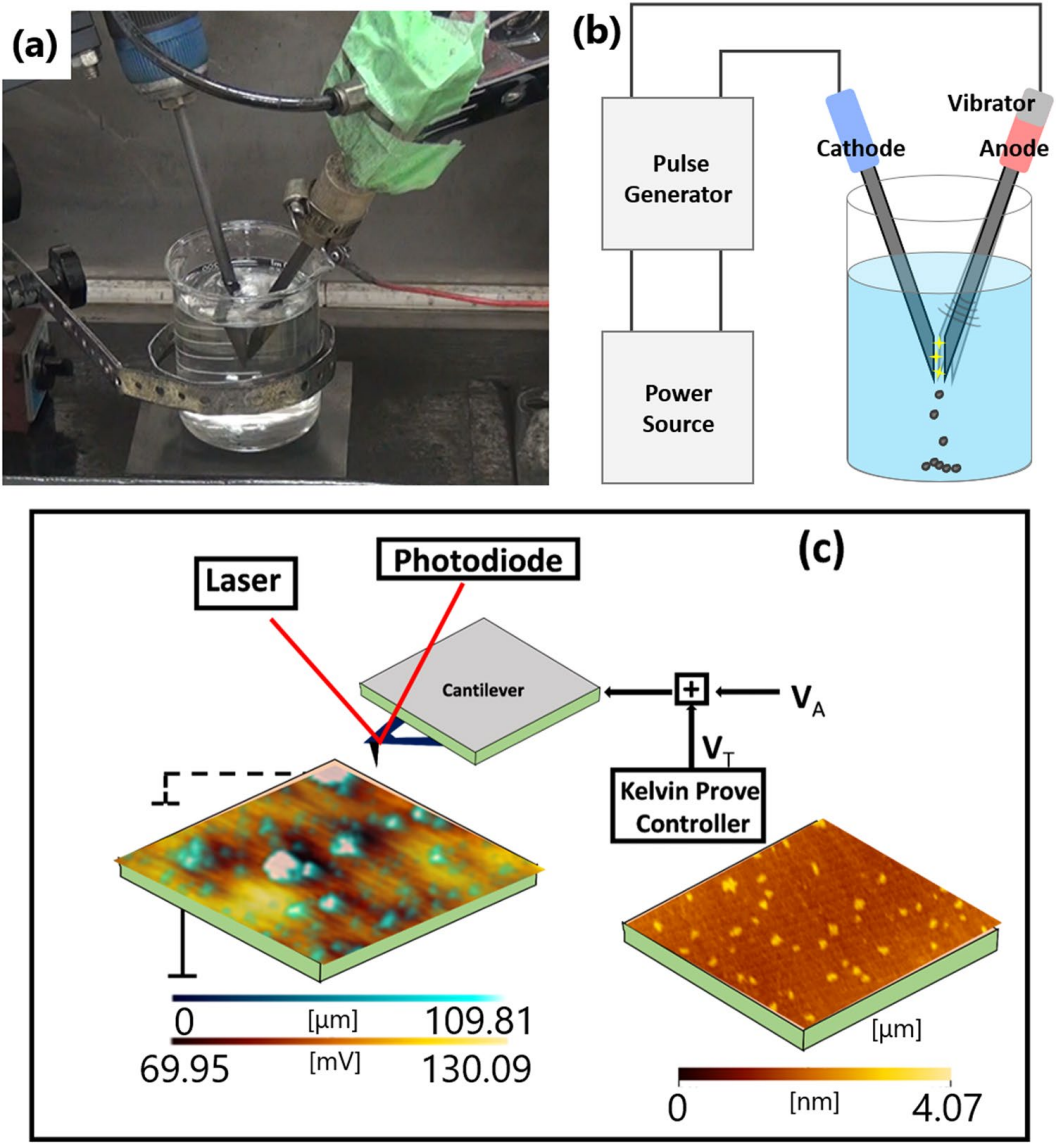
Photograph (**a**) and schematic (**b**) for the experimental setup of pulsed plasma treatment. Schematic illustration for the KPFM model used for investigating GG@CH_2_Cl_2_, GG@CHCl_3_ and GG@CCl_4_. VT presents the recorded contact potential. Brighter region indicates higher recorded contact potential difference. A lower VT represents a higher work function.

Simultaneous chlorination and exfoliation of G from graphite rod were accomplished by liquid pulsed plasma method in a glass made electrochemical cell, which was filled with the organochlorine solvent. The experimental setup is presented in Fig. 5. Highly pure graphite rod (diameter: 6 mm, 99.9998%) was submerged in the solvent and was treated by applying pulsed plasma (60 V, 2.5 A, 30 kHz, pulse duration: 10 µs) for 10 min. After the pulsed plasma treatment, the color of the clear solvent changed from limpid to black, which implied the formation of G colloid in the organochlorine solvent. Apparently, G seems to be exfoliated the graphite rod. A series of such suspension was prepared using CH_2_Cl_2_, CHCl_3_ and CCl_4_ as solvents. The G products were named as GG@CH_2_Cl_2_, GG@CHCl_3_ and GG@CCl_4_. In addition, a variety of solvents such as pure water, Ethanol (EtOH), Methanol (MeOH), Tetrahydrofurane (THF), Acetonitrile (ACN) and toluene were used also for observing the effect of solvent on exfoliation.

The morphology and thickness of the samples were studied by AFM (Bruker, Digital Instruments Nanoscope V). Raman spectroscopic investigations were performed on a micro Raman spectrometer (NRS-3100, Jasco, Japan) with a 532 nm excitation source at room temperature. An XPS instrument of Thermo Scientific, Sigma Probe was used for studying the chemical structure. A monochromatized X-ray source (Al Kα, hν = 1486.6 eV) and a discharge source (He I, hν = 21.2 eV) were used. Pt substrate was used to determine the Fermi level in Chlorinated G/Pt film. Vacuums better than 10^−7^ Pa was confirmed for the measurements. A hemispherical energy analyzer equipped with six channeltrons was used to detect the emitted electron. The KPFM measurements were accomplished at ambient conditions using a multimode microscope (Nanocute) operated by a Scanning Probe Microscopy controller. In the first step, along every scan line, the topography was recorded by controlling distance in tapping mode. In the second scan, the KFM measurement was performed using amplitude modulation (AM-KFM) with maintaining a constant distance from the sample (lift height = 5–50 nm). The applied electrical modulation frequency, V_A_, was adjusted to the eigenfrequency of the cantilever. Assumed that the Kelvin controller cancels electrostatic forces by adjusting the tip bias voltage, V_T_, until the amplitude becomes zero. The V_T_ reimburses the potential differences between sample spots and the probe tip. For a known value of tip work function ϴ_tp_, the work function of the sample can be calculated as ϴ_se_ = ϴ_tp_ − e ϴ_tp_. The variation in work function can be calculated to be independent of ϴ_tp_ as Δϴ_S_ = −eΔV_T_. Figure 5(c) represents the schematic for the measurement of V_T_ expressed in color codes with respect to the topography mapped along the z-axis. The V_T_ in fact represents the contact potential difference (CPD).

## Electronic supplementary material

Supplementary Information
