# Supplementary material for: Modulating the Work Function of Graphene by Pulsed Plasma Aided Controlled Chlorination
Source: Sci Rep. 2018 Nov 26;8:17392. doi: 10.1038/s41598-018-35668-x (PMC6255754; doi:10.1038/s41598-018-35668-x)
Supplement: Supplementary file 1 — Supplementary Information [file 41598_2018_35668_MOESM1_ESM.docx]

**Supplementary Information**

**Modulating the Work Function of Graphene by Pulsed Plasma Aided Controlled Chlorination**

Hiroshi Takehira, Mohammad Razaul Karim, Yuta Shudo, Masahiro Fukuda, Tsutomu Mashimo, and Shinya Hayami

Corresponding author:S. Hayami, hayami@kumamoto-u.ac.jp

**Supplementary Figures**


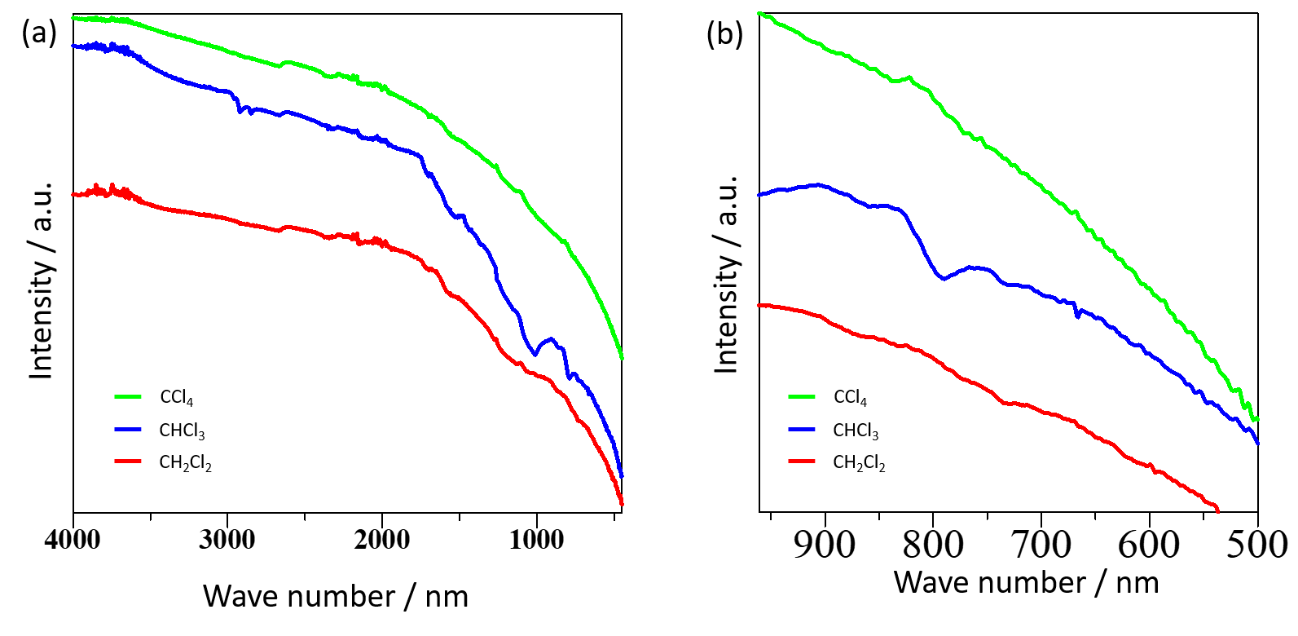


Figure S1. (a)over all and (b)enlarged FT-IR spectra for GG@CH_2_Cl_2_, GG@CCl_4_ and GG@CHCl_3_.


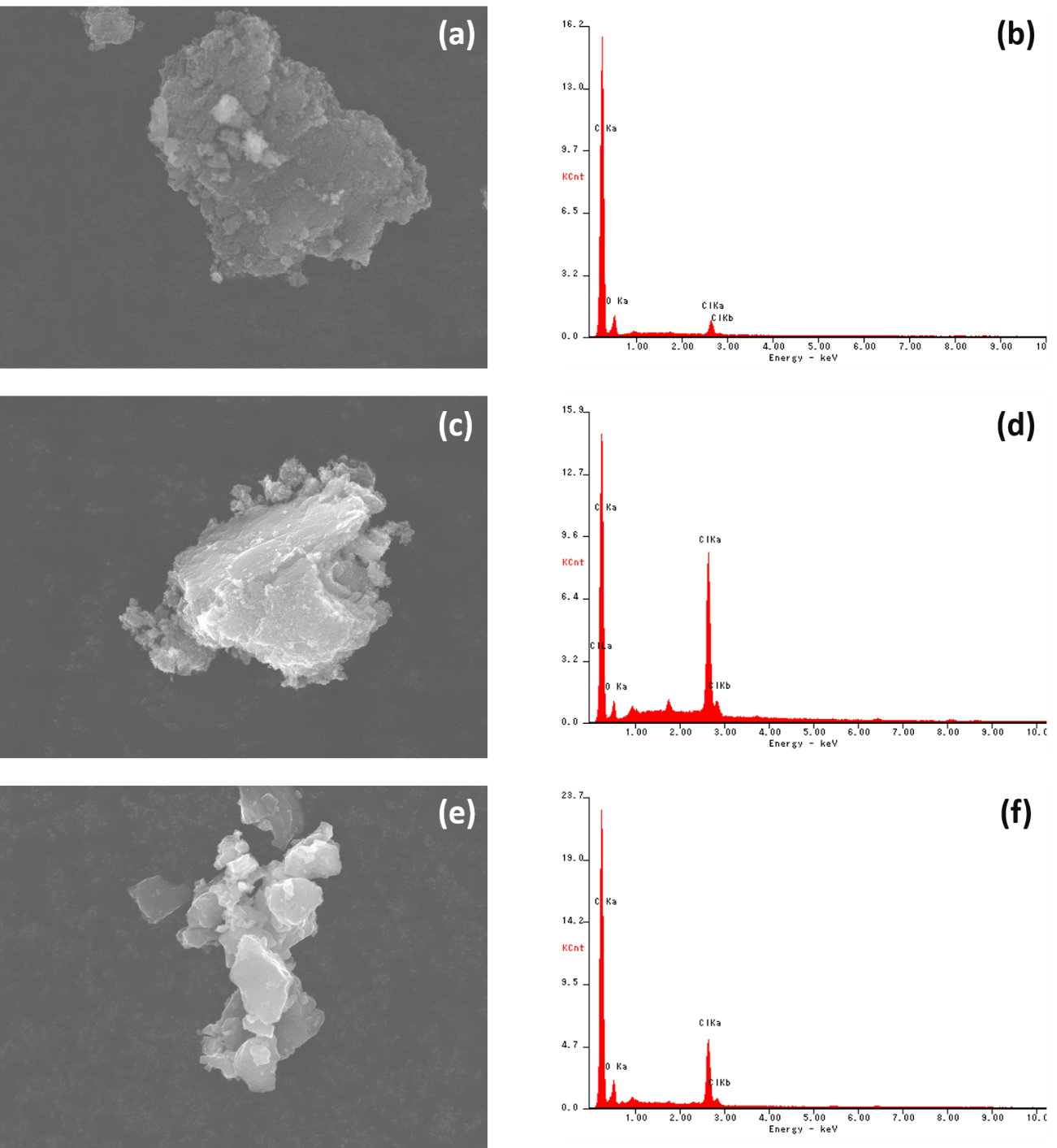


Figure S2. SEM image (a, c, e) and SEM-EDX spectra (b, d, f) of (a)GG@CH_2_Cl_2_ (a, b), GG@CCl (c, d) and GG@CHCl_4_ (e, f).


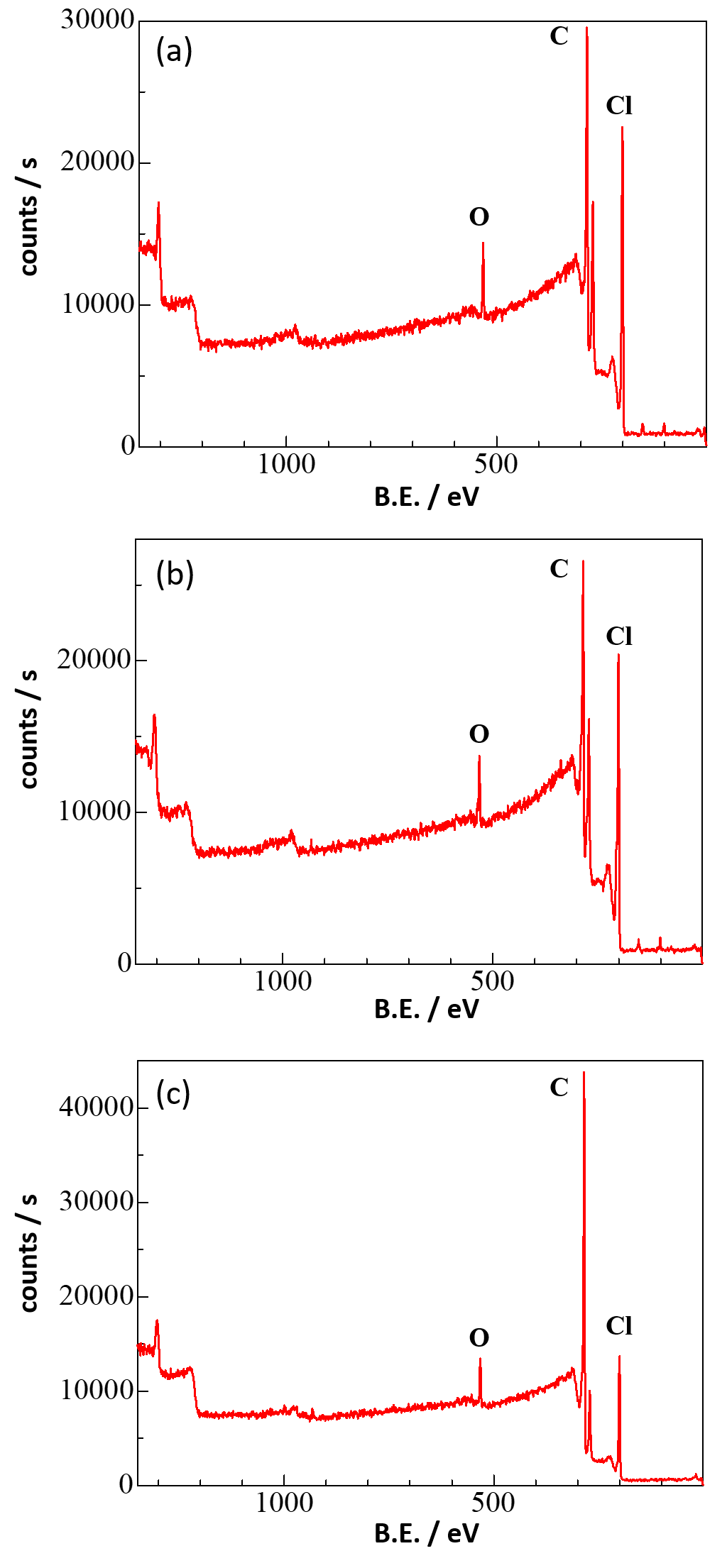


Figure S3. XPS spectra of (a)GG@CH_2_Cl_2_, (b)GG@CCl_3_ and (c)GG@CHCl_4_.
